# Supplementary material for: Ancient and Nonuniform Loss of Olfactory Receptor Expression Renders the Shark Nose a De Facto Vomeronasal Organ
Source: Mol Biol Evol. 2023 Mar 27;40(4):msad076. doi: 10.1093/molbev/msad076 (PMC10116579; doi:10.1093/molbev/msad076)
Supplement: msad076_Supplementary_Data [file msad076_supplementary_data.zip › SI fig.1 Olfactory receptor gene trees.pdf]

A - OR

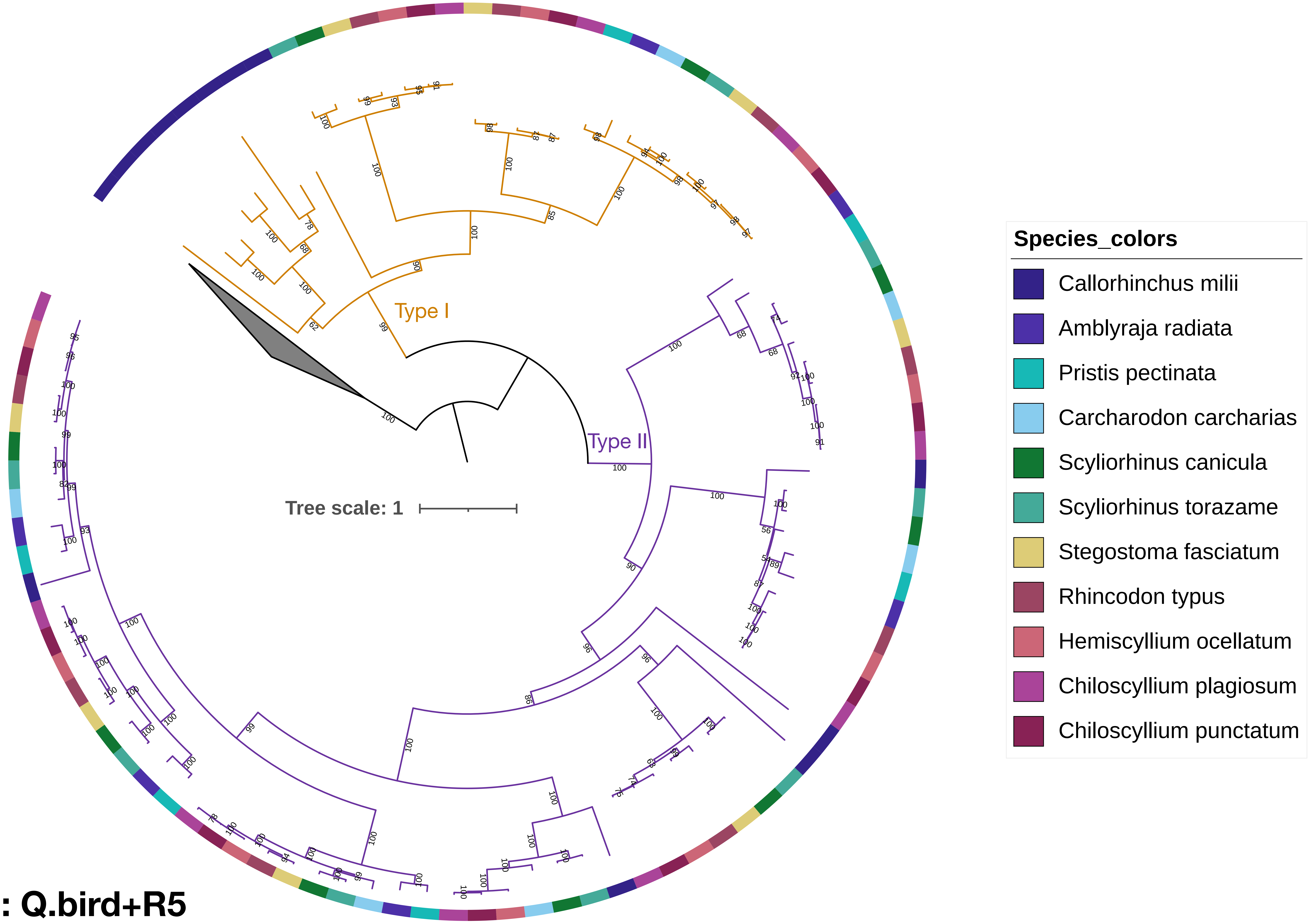

Substitution model : Q.bird+R5

B - ORA

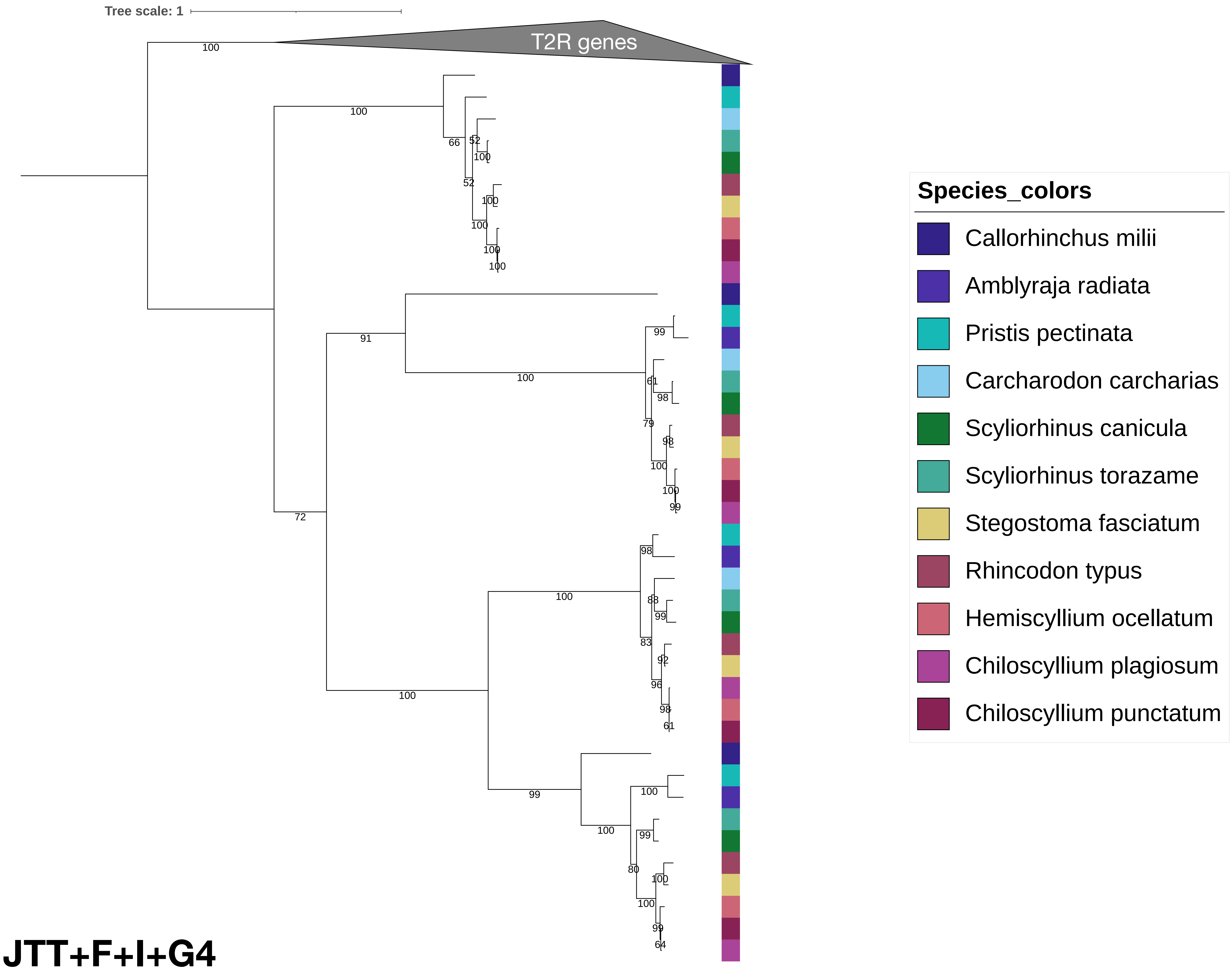

Substitution model : JTT+F+I+G4

C - TAAR + TARL

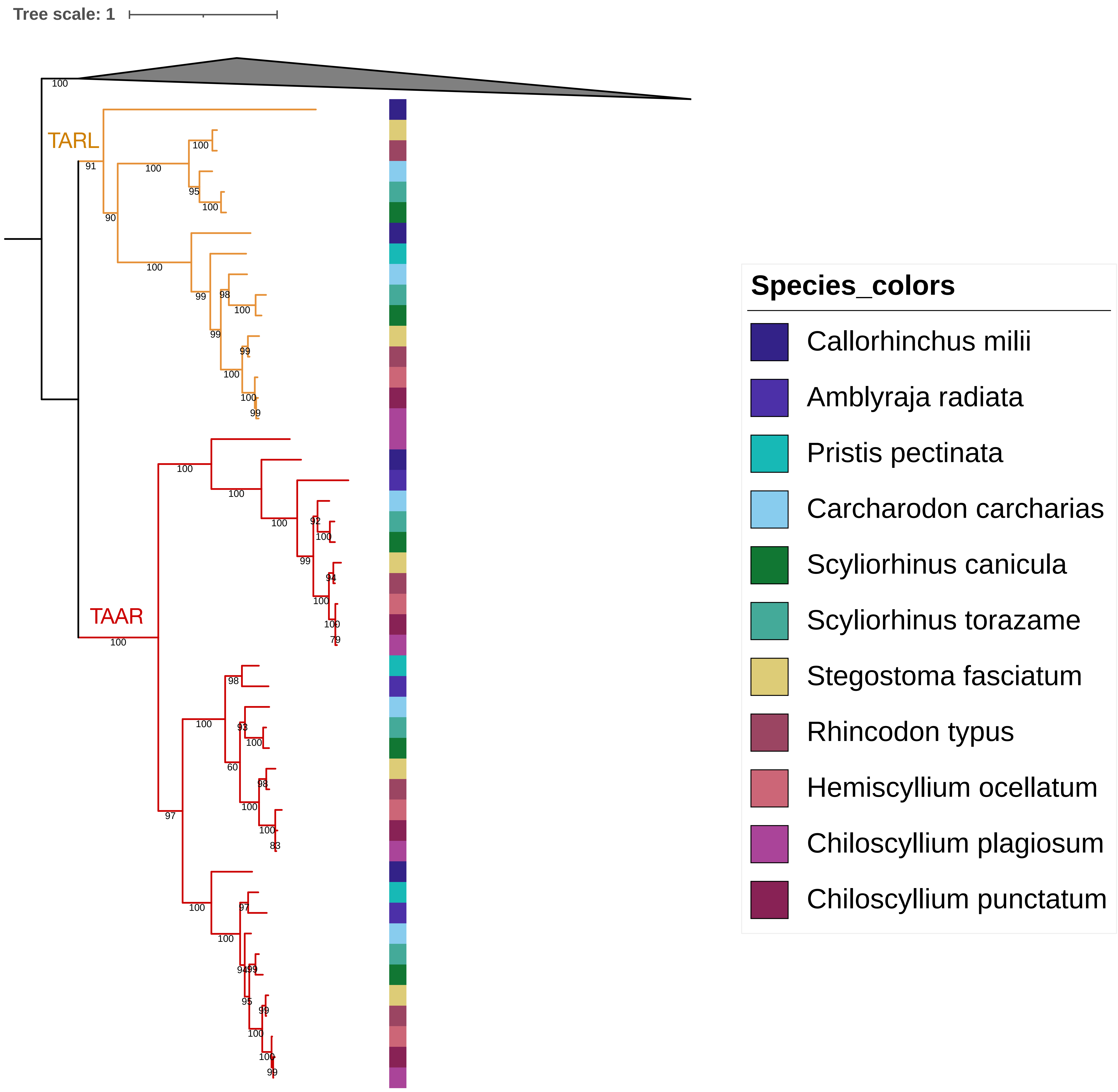

Substitution model : Q.plant+R5

D - V2R

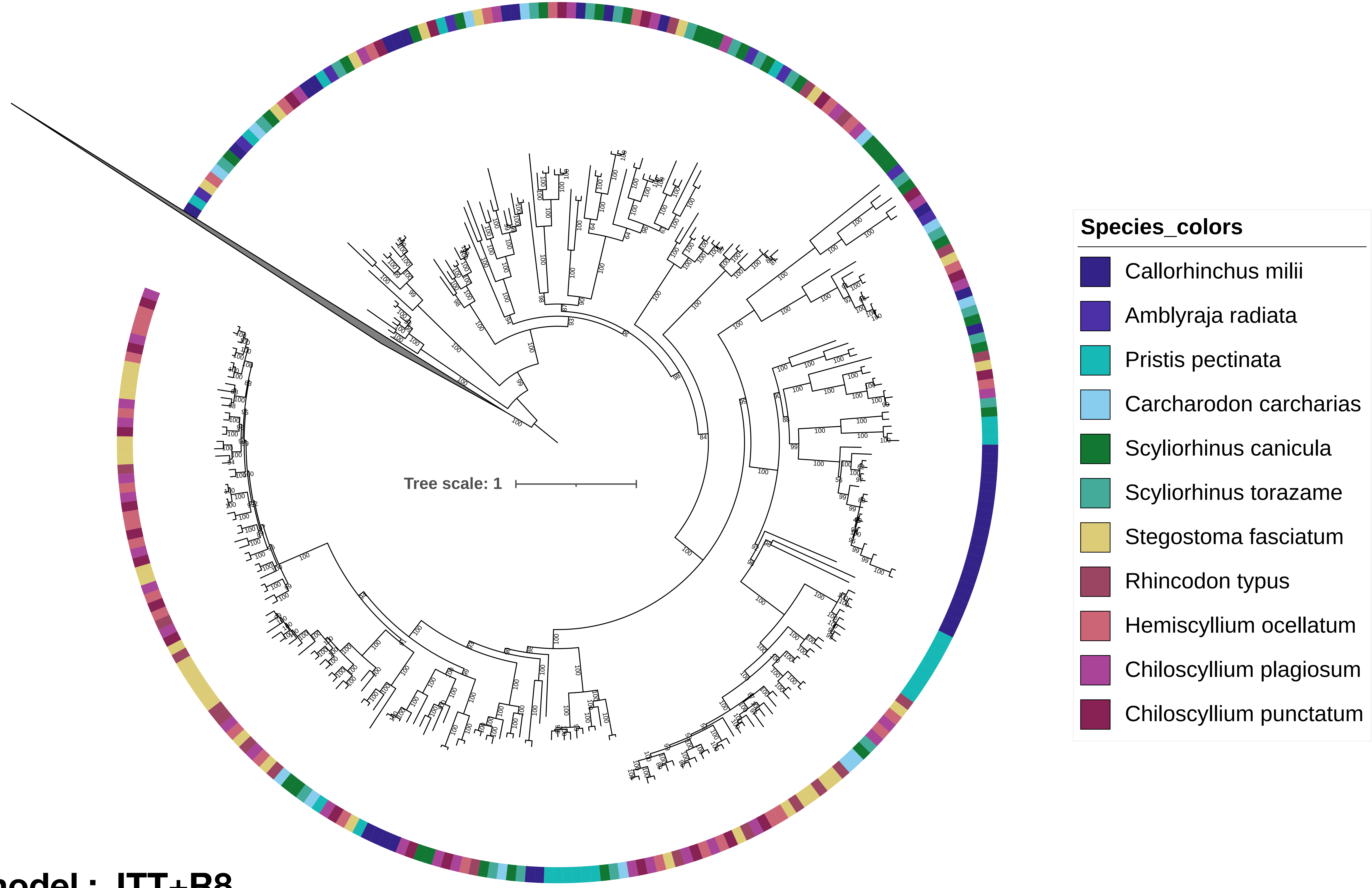

Substitution model : JTT+R8
