# Supplementary figures and images for: Ancient and Nonuniform Loss of Olfactory Receptor Expression Renders the Shark Nose a De Facto Vomeronasal Organ
Source: Mol Biol Evol. 2023 Mar 27;40(4):msad076. doi: 10.1093/molbev/msad076 (PMC10116579; doi:10.1093/molbev/msad076)

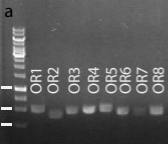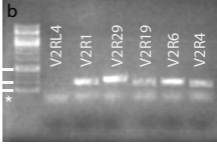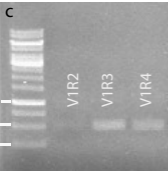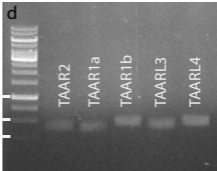

Supplement: msad076_Supplementary_Data [file msad076_supplementary_data.zip › Supplementary figure 2.pdf]

**A**

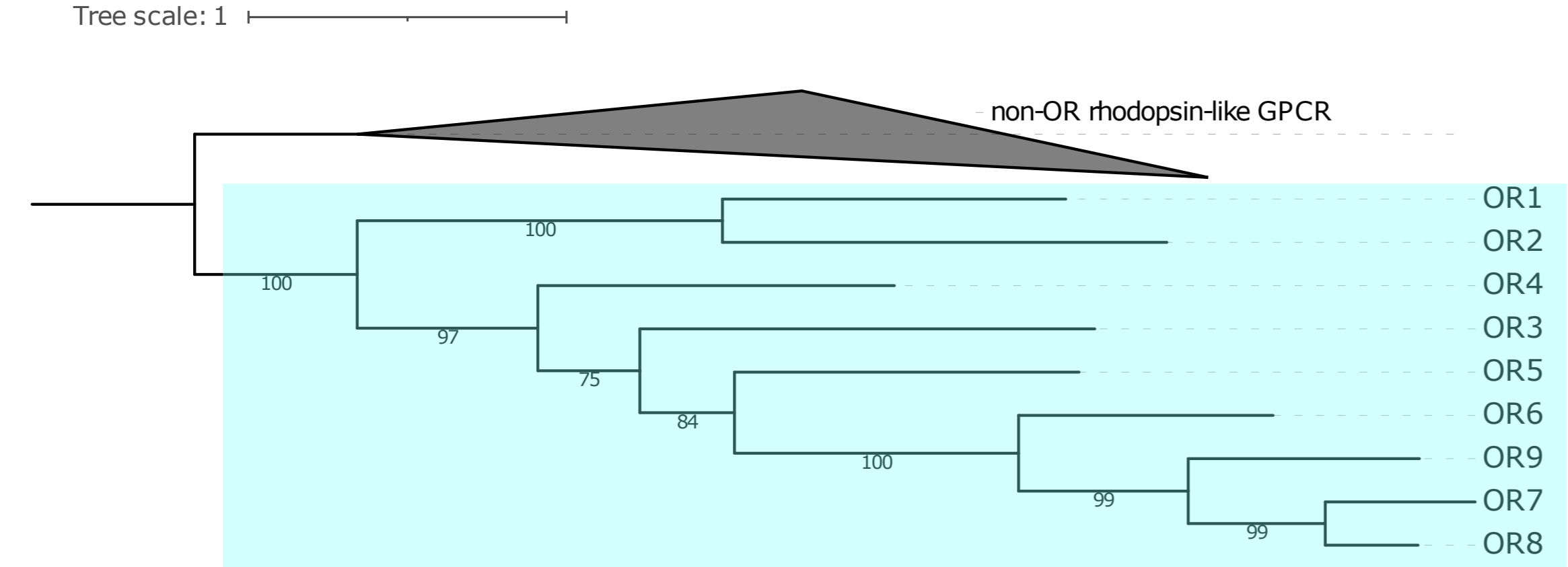

**B**

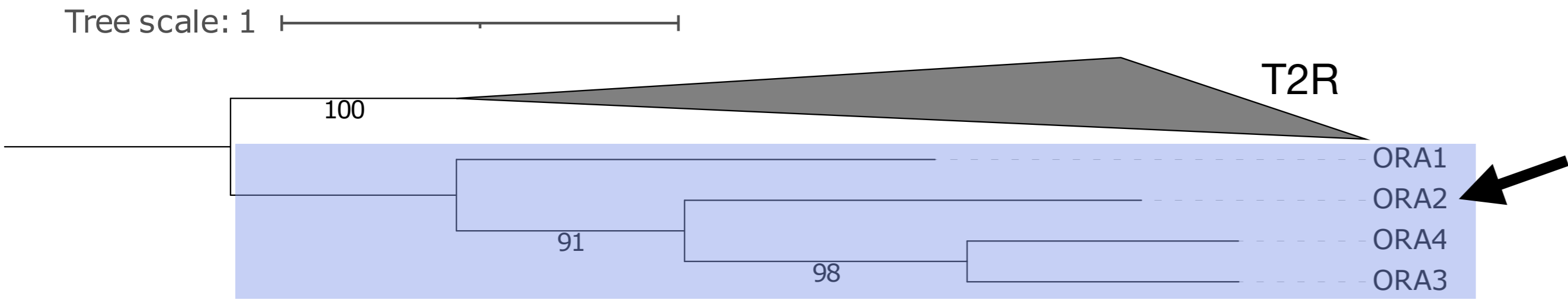

**C**

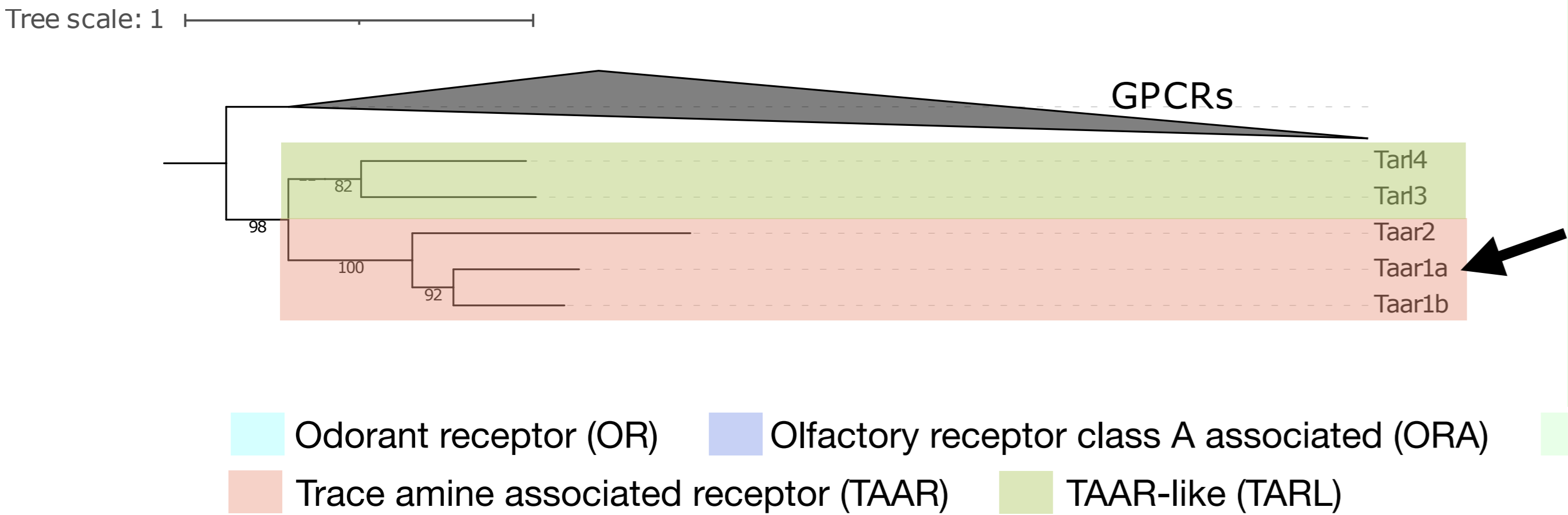

**D**

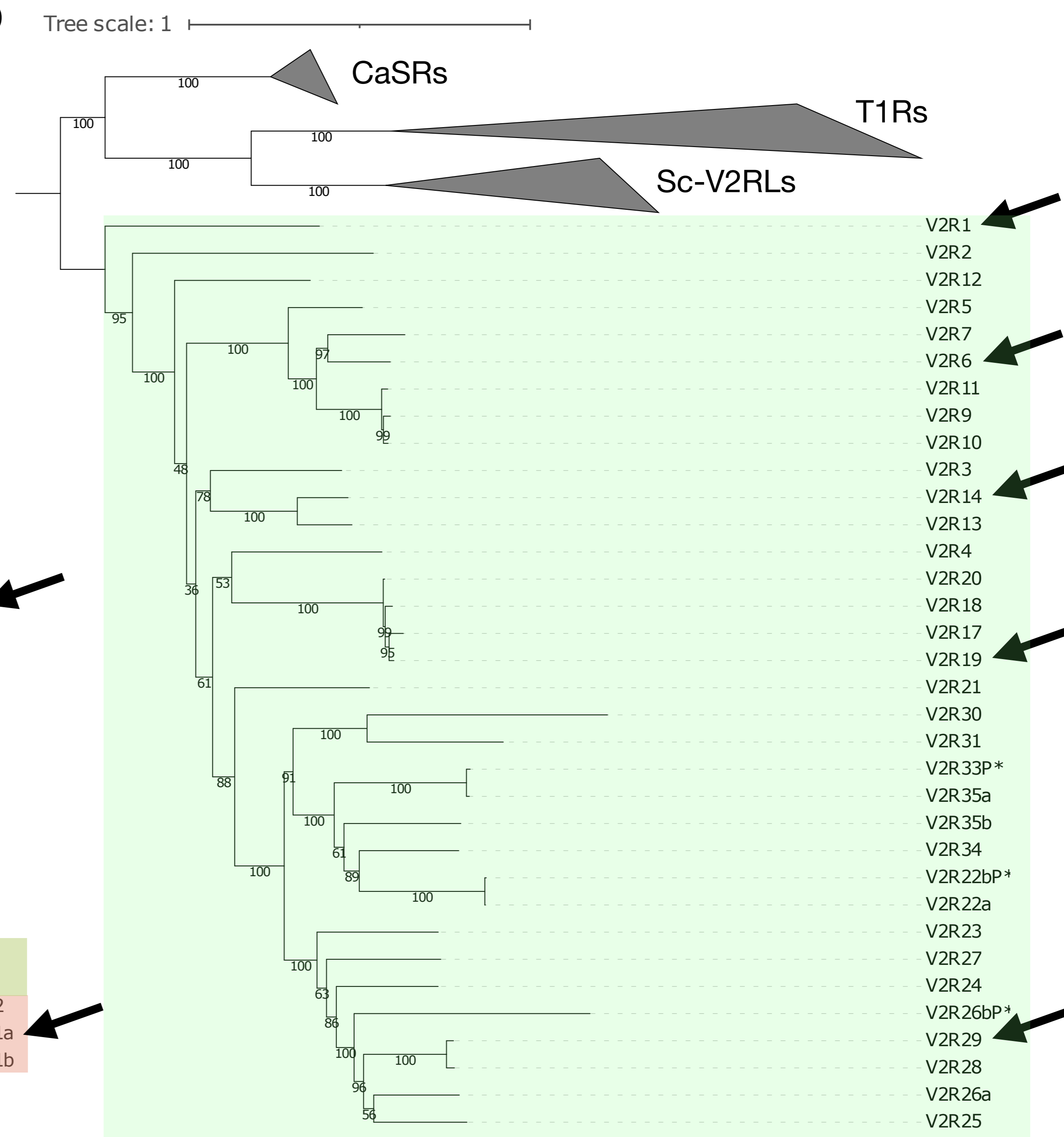

Supplement: msad076_Supplementary_Data [file msad076_supplementary_data.zip › SI fig.3 Catshark olfactory receptor repertoire with arrows for ISH polished.pdf]

# Expression of ORA2 and TAAR1a in OE

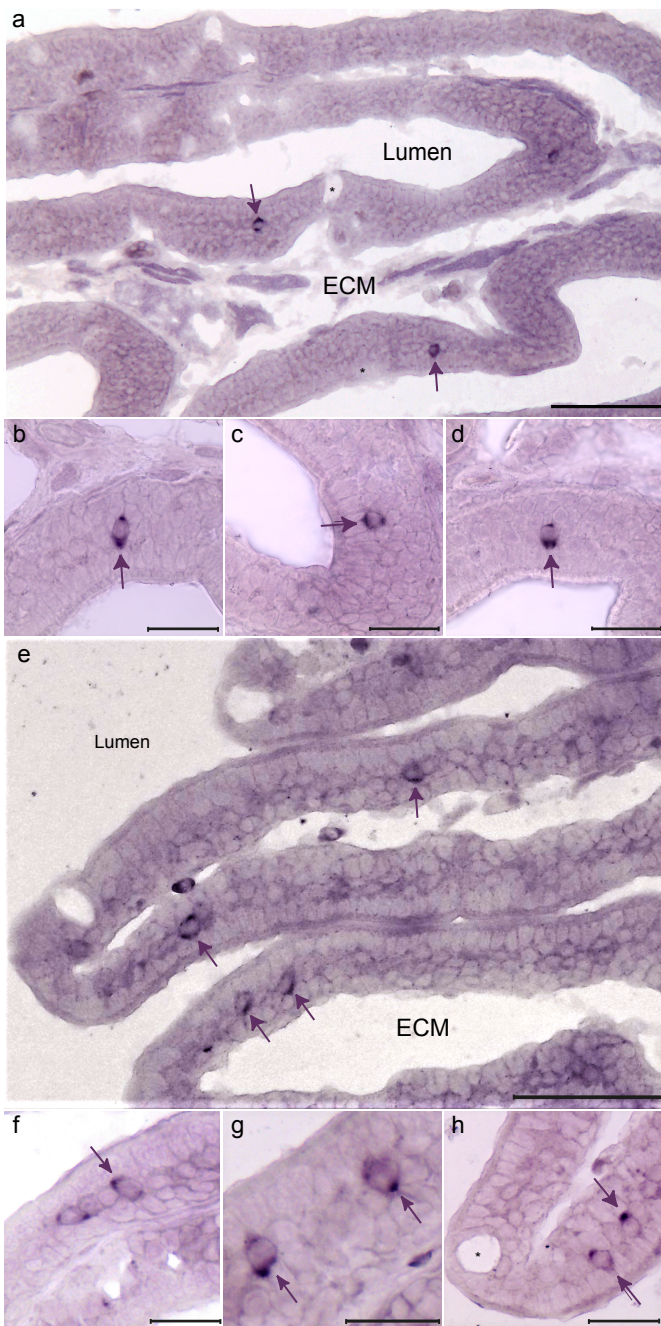

Supplement: msad076_Supplementary_Data [file msad076_supplementary_data.zip › SI fig.4 ORA2 und TAAR1a ISH.pdf]

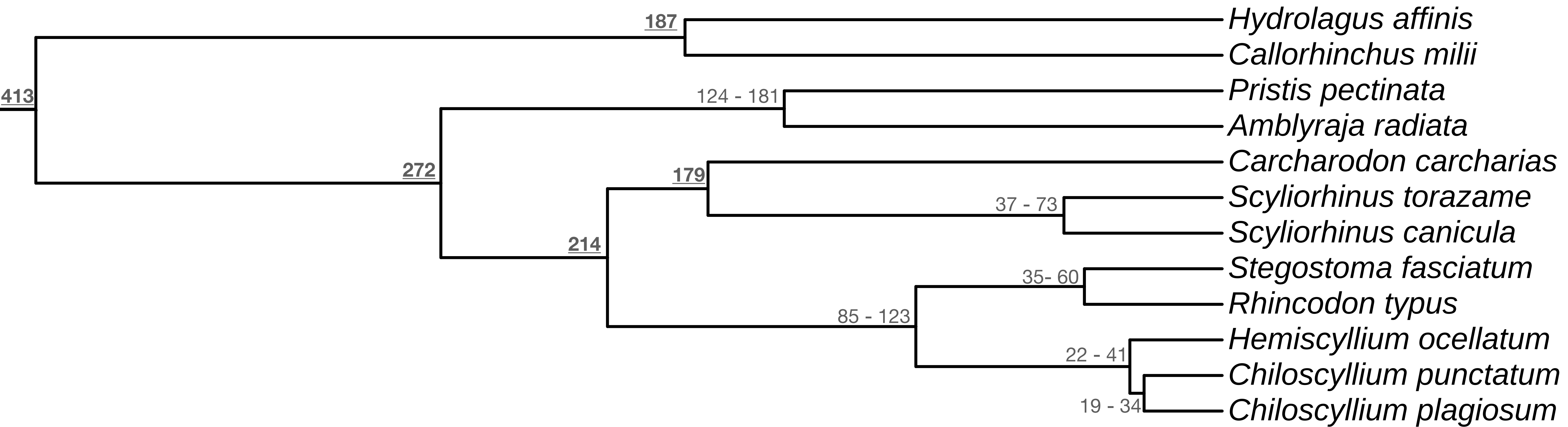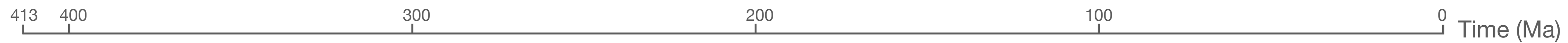

Supplement: msad076_Supplementary_Data [file msad076_supplementary_data.zip › SI fig.5 Complete species tree.pdf]

# BUSCO Assessment Results

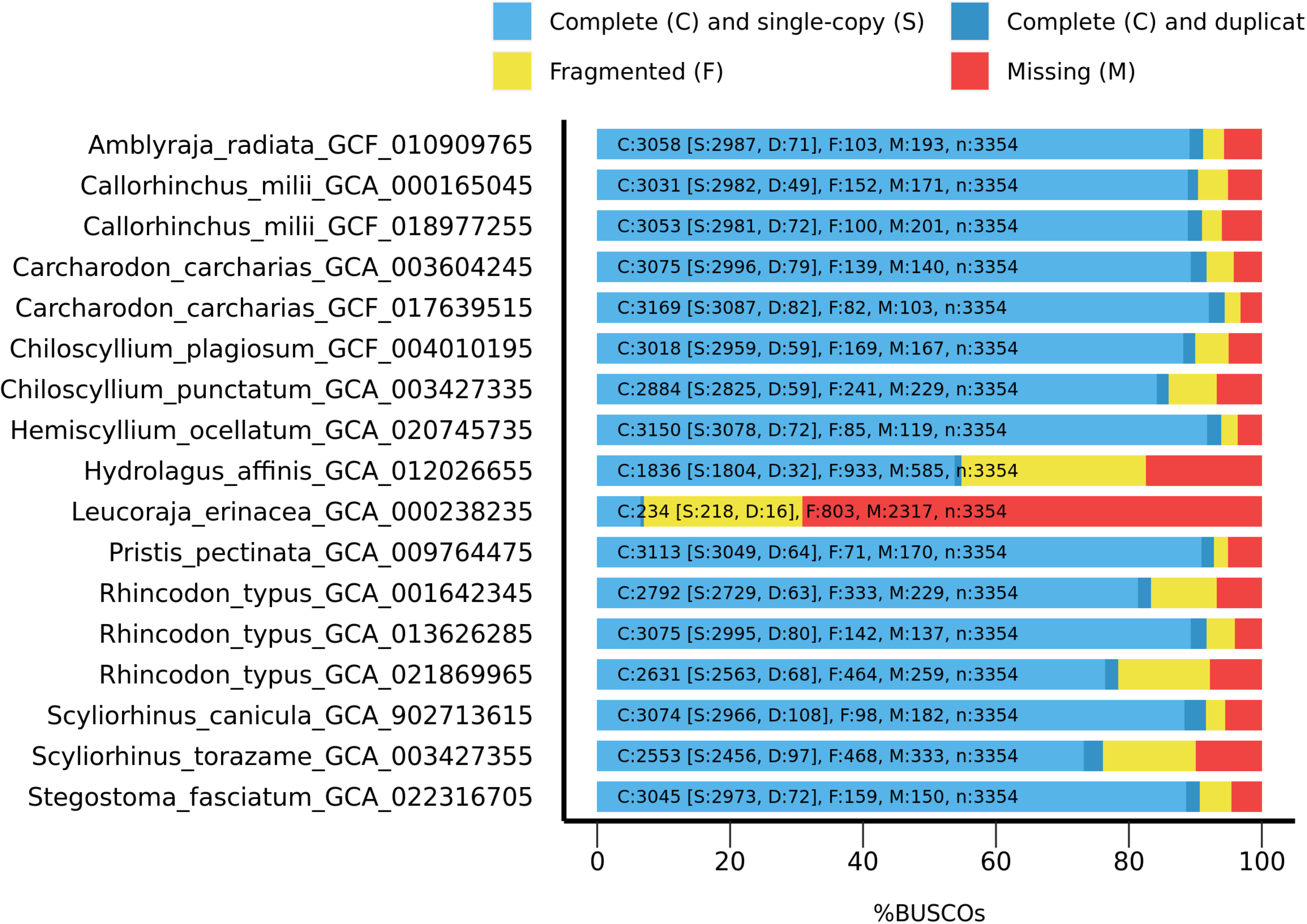

Supplement: msad076_Supplementary_Data [file msad076_supplementary_data.zip › SI fig.6 BUSCO results.pdf]
